# Selectively Cross-Linked Tetra-PEG Hydrogels Provide Control over Mechanical Strength with Minimal Impact on Diffusivity

**DOI:** 10.1021/acsbiomaterials.0c01723

**Published:** 2021-06-20

**Authors:** Suzette T. Lust, Dominique Hoogland, Michael D. A. Norman, Caoimhe Kerins, Jasmin Omar, Geraldine M. Jowett, Tracy T. L. Yu, Ziqian Yan, Jessie Z. Xu, Daniele Marciano, Ricardo M. P. da Silva, Cécile A. Dreiss, Pablo Lamata, Rebecca J. Shipley, Eileen Gentleman

**Affiliations:** † Centre for Craniofacial and Regenerative Biology, King’s College London, London SE1 9RT, United Kingdom; ‡ School of Biomedical Engineering and Imaging Sciences, King’s College London, London SE1 7EH, United Kingdom; § Department of Chemistry, King’s College London, London SE1 1DB, United Kingdom; ∥ Institute of Pharmaceutical Science, King’s College London, Franklin-Wilkins Building, London SE1 9NH, United Kingdom; ⊥ Institute of Healthcare Engineering and Department of Mechanical Engineering, University College London, London WC1E 7JE, United Kingdom

**Keywords:** hydrogel, diffusivity, mass transport, PEG, mesh size, rheology, diffusion modeling, obstruction theory

## Abstract

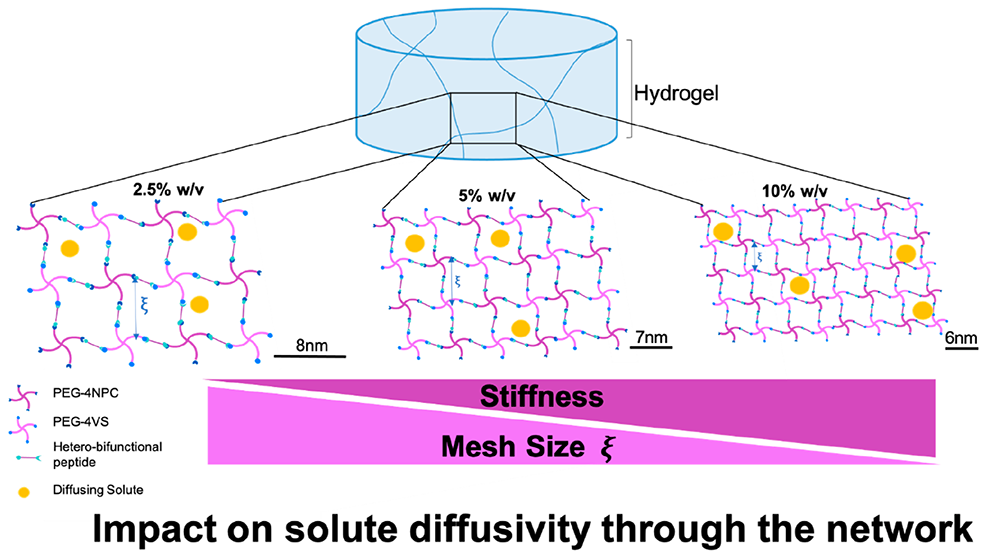

Synthetic hydrogels formed from poly(ethylene glycol) (PEG) are widely used to study how cells interact with their extracellular matrix. These *in vivo*-like 3D environments provide a basis for tissue engineering and cell therapies but also for research into fundamental biological questions and disease modeling. The physical properties of PEG hydrogels can be modulated to provide mechanical cues to encapsulated cells; however, the impact of changing hydrogel stiffness on the diffusivity of solutes to and from encapsulated cells has received only limited attention. This is particularly true in selectively cross-linked “tetra-PEG” hydrogels, whose design limits network inhomogeneities. Here, we used a combination of theoretical calculations, predictive modeling, and experimental measurements of hydrogel swelling, rheological behavior, and diffusion kinetics to characterize tetra-PEG hydrogels’ permissiveness to the diffusion of molecules of biologically relevant size as we changed polymer concentration, and thus hydrogel mechanical strength. Our models predict that hydrogel mesh size has little effect on the diffusivity of model molecules and instead predicts that diffusion rates are more highly dependent on solute size. Indeed, our model predicts that changes in hydrogel mesh size only begin to have a non-negligible impact on the concentration of a solute that diffuses out of hydrogels for the smallest mesh sizes and largest diffusing solutes. Experimental measurements characterizing the diffusion of fluorescein isothiocyanate (FITC)-labeled dextran molecules of known size aligned well with modeling predictions and suggest that doubling the polymer concentration from 2.5% (w/v) to 5% produces stiffer gels with faster gelling kinetics without affecting the diffusivity of solutes of biologically relevant size but that 10% hydrogels can slow their diffusion. Our findings provide confidence that the stiffness of tetra-PEG hydrogels can be modulated over a physiological range without significantly impacting the transport rates of solutes to and from encapsulated cells.

## Introduction

Cells’ interactions with their local environment are known to play central roles in regulating processes including proliferation, migration, differentiation, and phenotypic maintenance.^1−3^ By extension, these interactions are also involved in dysregulation of cell behavior in pathologies. Thus, understanding the impact of mechanical and biological cues cells receive from their surroundings is key in both disease modeling and the development of regenerative therapies.^4,5^ While the ability of whole organisms and tissue explants to provide physiologically relevant environments to cells are unrivalled, there is also a need for simpler reductionist models that allow for studies into how specific cues impact cellular behaviors. Such models have the potential to identify underlying mechanisms that govern complex tissue pathologies, can reveal fundamental insights into cell-matrix interactions, and may inform methods to engineer tissues for regenerative applications.

While *in vitro* cell cultures have revolutionized our understanding of mammalian biology, cells respond differently when within 3D structures akin to tissues compared to on 2D surfaces.^6,7^ Indeed, among other factors, the transport of molecules to and from cells is markedly changed in 3D. Hydrated polymer networks called hydrogels can mimic many aspects of the 3D environment cells inhabit *in vivo*. Their compatibility hinges on their two-phase nature, with a solid polymer scaffold mimicking the extracellular matrix (ECM) and the liquid phase available for transport of nutrients.^8^ Moreover, the properties of the hydrogel network can be tuned to mimic characteristics of the native tissue, including their stiffness, which is known to regulate a range of cellular behaviors, including fate specification.^1−3^

Synthetic hydrogels formed from poly(ethylene glycol) (PEG) are suitable for cell encapsulation due to PEG’s stability, hydrophilicity, and resistance to protein adsorption.^9^ Furthermore, the versatility with which PEG macromers can be cross-linked allows control over theoretical mesh size by simply changing polymer concentration, macromer arm size, and the number of arms. And, while native biochemical cues are missing in PEG hydrogels, the polymer can be modified to include ECM-mimicking anchorage sites. Furthermore, cross-linking the network with matrix metalloproteinase (MMP)-sensitive peptides allows encapsulated cells to actively remodel and migrate through them.^10,11^ However, the introduction of bioactive motifs often leads to network inhomogeneities.^12^ These irregularities are caused by missing cross-links, internal loops within individual polymer macromers, and dangling polymer ends.^13,14^ Such inhomogeneities, although potentially useful as means to permit diffusion, can lead to reduced stiffness.^15,16^ Moreover, as inhomogeneities push gel structures further from the ideal network, theoretical characterizations fall short, making predictions of hydrogel properties more complex and attributing them to biological outcomes more fraught.^17^ Therefore, hydrogel designs that reduce inhomogeneities may provide a more effective and controlled platform for studying cellular behaviors in 3D.

Many covalently cross-linked hydrogel networks rely on Michael-type additions between a cysteine residue at the end of a peptide and an alkene-containing end group on the PEG macromer arm (either 4-arm or 8-arm, B_4_/B_8_). Peptide sequences susceptible to enzymatic degradation are then created with cysteine groups at both termini (A_2_), creating A_2_+B_4_/B_8_ designs. In such designs, homobifunctional cross-linking peptides react with the polymer chain ends indiscriminately. In this scenario, primary loops in which one peptide reacts at both ends on the same macromer are likely to form, particularly at low polymer concentrations. Adhesive motifs, on the other hand, typically have a single cysteine group, and thus are incorporated in a pendant fashion. In the latter arrangement, as more pendant groups are introduced, the number of arms available for cross-linking is reduced, increasing gel inhomogeneities.

To circumvent these issues, it is possible to selectively functionalize end groups of both the polymer backbone and peptides, ensuring that each can only react in a desired manner. Indeed, the Shibayama group has reported on highly homogeneous, high-strength “tetra-PEG” hydrogels that form upon mixing two polymer macromers with different reactive terminal groups (A_4_+B_4_).^15^ We hypothesized that it would also be possible to create efficiently cross-linked A_4_+B_4_ hydrogels suitable for supporting live cells. However, the implications of the A_4_+B_4_ design on mass transport to and from encapsulated cells has not been investigated thoroughly.

To create A_4_+B_4_/tetra-PEG hydrogels, we created heterobifunctional peptides and reacted an amine at the peptides’ N-terminus with nitrophenyl carbonate (NPC) end-functionalized four-arm PEG (PEG-4NPC, A_4_), creating PEG–peptide conjugates. We then formed hydrogels by reacting a free thiol from a cysteine residue located at the peptides’ C-terminus with vinyl sulfone (VS) end-functionalized four-arm PEG (PEG-4VS, B_4_). We have previously shown that when adhesive (RGD) and MMP-degradable peptide sequences are used to cross-link the PEG network, this design supports the viability of encapsulated human induced pluripotent stem cell-derived intestinal organoids.^11^ Importantly, even within these soft matrices (elastic modulus, ∼ 1 kPa), gelation was quick enough that organoids did not fall to the bottom of the hydrogel prior to gelation, suggesting that network formation was more effective at polymer concentrations as low as 2.5% compared to similar A_2_+B_4_ designs.^18^

The tetra-PEG design allows physical and biological properties of the hydrogel to be tuned independently, while maintaining network connectivity. Indeed, as MMP-susceptible and adhesive peptides both participate in cross-linking, cellular response to mechanical stiffness can be studied without altering adhesiveness or degradability. However, at higher polymer concentrations, the space between cross-links in the polymer phase, known as the mesh size, is reduced. It therefore follows that higher polymer concentrations may not only change cells’ mechanical environment but also impact the mass transport of solutes. Indeed, others have shown that for some hydrogel systems, increasing polymer concentration impacts diffusivity.^19,20^ For both *in vitro* models and regenerative applications, the ability of nutrients to reach encapsulated cells over a reasonable time scale is crucial. Diffusivity will also impact researchers’ ability to detect secreted molecules in the culture supernatant, which may be of interest for monitoring cell behaviors. Moreover, time scales for diffusion of biomolecules can impact cell–cell communication,^21,22^ which may play a role in regulating autocrine versus paracrine signaling effects.

Here, we combined predictive models with experimental characterization to study how altering polymer concentration in tetra-PEG hydrogels impacts the network’s permissiveness to the diffusion of molecules. Our findings show that hydrogel stiffness can be modulated over a large range while only impacting diffusivity negligibly, as we only observed significant changes in diffusion at high polymer concentrations that are less suitable for encapsulating cells.

## Materials and Methods

### PEG–Peptide Conjugate Synthesis and Hydrogel Formation

**Figure 1:**
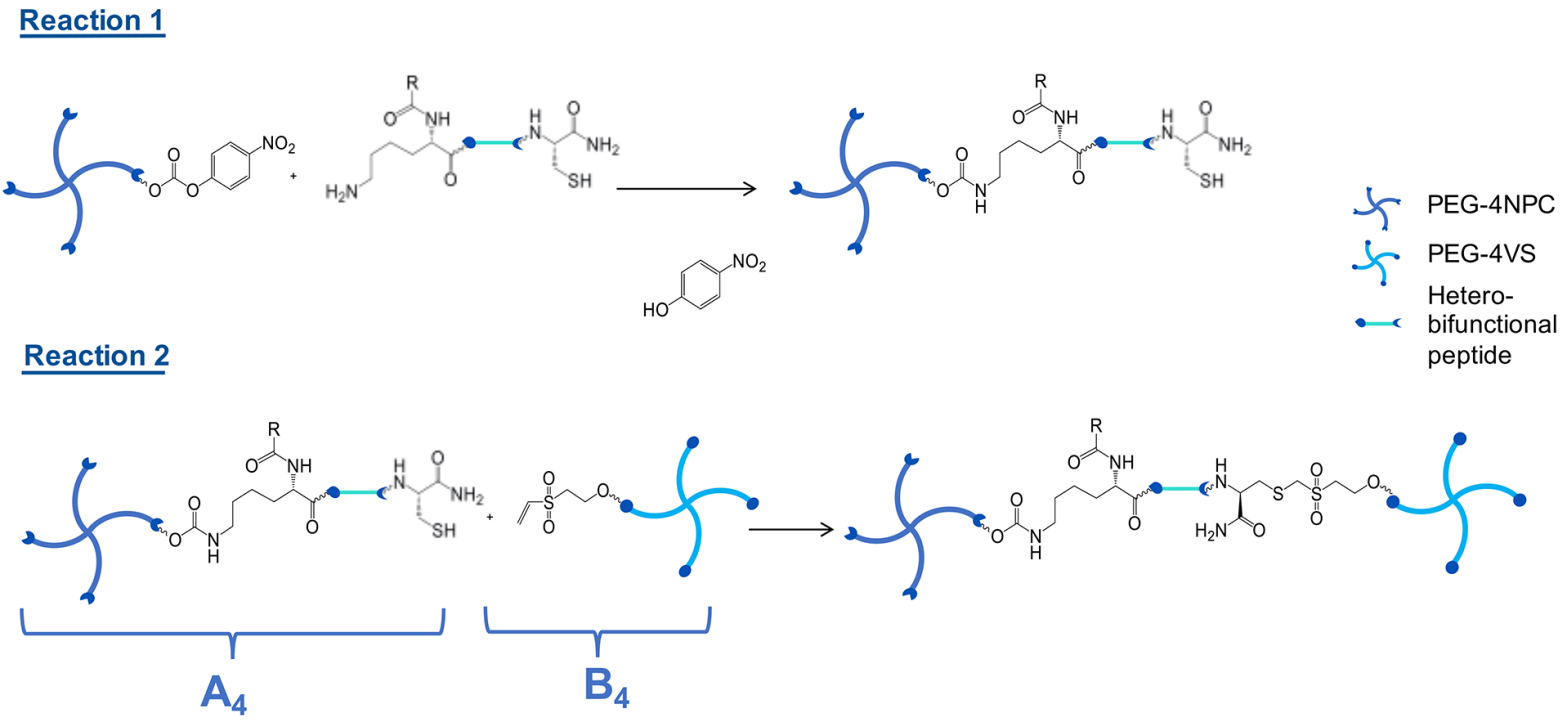
Diagram of the reaction schemes used to form hydrogels. Reaction 1 conjugates peptides with PEG-4NPC. Reaction 2 selectively cross-links PEG-peptide conjugates (A_4_) with PEG-4VS (B_4_) using a Michael-type addition.

PEG–peptide conjugates were synthesized as described previously.^11^ Briefly, peptide Ac-KDW-ERC-NH2 (custom synthesis Peptide Protein Research, Ltd. (UK), >98% purity) with an N-terminal primary amine (lysine side chain) and C-terminal thiol (cysteine) were reacted with a four-arm 10 kDa PEG-NPC (JenKem Technology, USA) to form PEG–peptide conjugates (Figure 1). Twenty or 15 μL (depending on end volume) of purified conjugate solution was then cross-linked with 20 or 15 μL of 10 kDa PEG tetramer solution with a vinyl-sulphone end group (JenKem Technology, USA) at the required concentrations at 37 °C through a base-catalyzed (pH 8) Michael-type addition. This strategy was used to make 2.5%, 5%, and 10% (w/v) hydrogels.

### Swelling

First, 30 μL hydrogels were formed in Sigmacote (Sigma UK)-treated 6-mm-diameter glass cylindrical molds and submerged in PBS. Hydrogel wet weight was measured once swelling equilibrium had been achieved (after 48 h). Hydrogels were lyophilized to determine dry weight and the mass swelling ratio calculated using
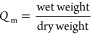
1

The mass swelling ratio was then used to calculate the volumetric swelling ratio *Q*_v_ using the following relation:
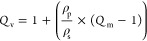
2where ρ_p_ and ρ_s_ are the polymer and solvent densities, respectively. This parameter describes the amount of water within the hydrogel in the swollen state and can be used to infer network connectivity. Data were analyzed for significance using one-way Anova with Tukey’s multiple comparison test.

### Rheological Measurements of Hydrogel Gelation and Mechanical Properties

Hydrogel gelation was assessed on a strain-controlled ARES from TA Instruments using a 25 mm cone with a 0.02-rad angle and plate by carrying out small-amplitude oscillatory time-sweep measurements at a strain of 5% and a constant angular frequency of 1 rad s^–1^. All measurements were carried out at 37 °C, depositing paraffin oil on the edges of the sample to prevent evaporation. To perform measurements, 86 μL of hydrogel precursor solution was placed in the instrument, and storage modulus *G*′ and loss modulus *G*″ were recorded as a function of time (Orchestrator software, version 7.2.0.2). Subsequently, an amplitude sweep was carried out, recording *G*′ and *G*″ over the range 1–100% shear strain for 2.5 and 5% gels and 1-25% for the 10% gel, at a fixed frequency of 1 rad s^–1^; this range was found to be within the linear viscoelastic region. Finally, a frequency sweep was recorded, measuring *G*′ and *G*″ as a function of shear frequency in the range 100–0.1 rad s^–1^ at a fixed strain of 5%. To assess whether storage and loss moduli were significantly different between samples with varying polymer concentration, a one-way ANOVA with Tukey’s multiple comparison correction was performed.

### Theoretical Estimations of Mesh Size

Hydrogels can be modeled as polymer strands that cross-link to form a network, where mesh size, ξ, is the distance between cross-links. In this scenario, ξ is the size of the spaces between polymer chains through which liquid and solutes can move. Therefore, mesh size influences the diffusivity of the network and the time scales taken to reach equilibrium for any solute diffusing through it. It can be challenging to directly measure mesh size without dehydrating the polymer network.^23,24^ Therefore, theoretical estimates are obtained by estimating the molecular weight between cross-links and thus the length of these chains. Mesh size can be estimated using a variety of experimental techniques including dynamic light scattering, small angle neutron scattering, and small-angle X-ray scattering.^25^ However, because of experimental limitations, it is most often estimated using simpler methods based on experimental parameters gathered from (1) rheological data, (2) swelling data, and (3) direct measurements of diffusivity.^24^

We make our estimates for network size here using swelling data. We consider ξ as the average distance between cross-links, and hence a measure of the distance between two adjacent polymer strands in a hydrogel network in its equilibrium swollen state. Equilibrium swelling theory balances the thermal energy due to interactions between polymer and liquid molecules and the elastic tension in the polymer arms in the swollen state.^24,26^ This relation has been shown to faithfully predict PEG gel swelling^27^ and has been used previously to calculate mesh size in hydrogels used for *in vitro* cell cultures.^28−30^ The Flory–Rehner theory states that the change in the potential energy in the system during transition from its preswollen to swollen state is equal to the increase in elastic forces in the system and that these two terms are equal at equilibrium. Therefore, when the expressions for the thermal mixing energy and the elastic energy in the polymer strands are used, the average molecular weight between cross-links *M̅*_c_ in g/mol is given by
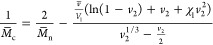
3where *M̅*_n_ is the molecular weight of the polymer chains in the absence of the cross-linking agent in g/mol, *v̅* is the specific volume of the polymer and is defined as the ratio of polymer density to solute density (dimensionless), *V*_1_ is the molar volume of the solvent in cm^3^/mol,^31^*v*_2_ is the polymer volume fraction in the swollen state (reciprocal of *Q*_v_ obtained from swelling measurements) (dimensionless), and χ_1_ is the polymer–solvent interaction parameter (a measure of the degree of interaction between the polymer chains and the surrounding solution; dimensionless). Methods for determining these parameters, and in particular that for χ_1_, have improved understanding of the swelling equilibrium in hydrogels.^24^ The values for each parameter for the A_4_+B_4_ hydrogels examined in this study are given in Table 1.

**Table 1:** Parameters Used in Theoretical Mesh Size Estimations

parameter name	explanation	value
*M̅*_n_	molecular weight of the polymer chains in the absence of the cross-linking agent	2.94 × 10^3^ [g/mol]
*v*	specific volume of the polymer	0.9
*V*_1_	molar volume of water	18 [cm^3^/mol]
*V*_2_	polymer volume fraction in the swollen state (reciprocal of *Q*_v_ obtained from swelling data)	0.024 for 2.5% hydrogels, 0.034 for 5% hydrogels, and 0.051 for 10% hydrogels
*X*_1_	the polymer solvent interaction parameter	0.426^49^
*l*	average bond length between repeating units	0.146 [nm]^32^
*C*_n_	Flory characteristic ratio	4^32^
*M*_r_	molecular weight of repeating unit	44 [g/mol]^32^

Once the average molecular weight between cross-links is found, this must be converted to an estimate of the end-to-end distance to predict ξ. The end-to-end distance between two adjacent cross-links before the network is stretched is given by  and obtained in nanometers using
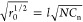
4in which *l* is the average bond length between repeating units in nanometers in the polymer chain, *N* is the number of links between monomers in the chain (dimensionless), and *C*_n_ is the Flory characteristic ratio (dimensionless value of 4 for long PEG chains).^24,32^ This parameter *C*_n_ is defined as the ratio of polymer chain length to the theoretical length when each section is considered to be freely jointed and can be randomly oriented with no influence from external forces, taking into account steric interference (nonbonded interactions between molecules). Further, *N* is given by
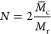
5where *M*_r_ is the molecular mass of the repeating unit in g/mol.^33^

When the polymer chain becomes stretched at the swelling equilibrium, the end-to-end distance is increased in the direction of the net stretching force.^34^ A measure of this distance increase is given by the elongation ratio, which is the ratio of the new polymer chain length once the force has been applied to that of the unstretched end-to-end distance. This ratio is approximated for polymers which swell in all directions equally (isotropic).^26^ The mesh size can then be expressed as
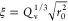
6

### Modeling Solute Diffusion out of the Hydrogel

We set up a diffusion model to simulate transport of solutes out of our tetra-PEG hydrogels and into the surrounding solution to assess the impact of mesh size on the diffusivity of molecules of relevant size. The model was established to mimic a standard experimental setup in which molecules of known size diffuse from hydrogels. This allowed for direct comparison between our theoretical predictions and experimental measurements.

Diffusion of solutes through a hydrogel is hindered by the presence of the polymer chains creating a mesh through which the solutes must move. This diffusibility is therefore determined by the relative size of the solute compared to the mesh size, the mobility of the polymer chains, and the potential steric interactions between solutes and polymer chains.^19^ Models have been reported that predict solute transport through hydrated polymer networks with differing emphasis on the main obstruction mechanism.^19,24^ We represented the solute molecules by hard spheres. The polymer chains were assumed to be immobile, and we neglected steric interactions between these and the solute. In such models, the presence of the polymer phase results in an increased path length for diffusing molecules slowing their transport. Similar models have been shown to effectively predict mass transport phenomena in hydrogels.^19,35^

**Figure 2:**
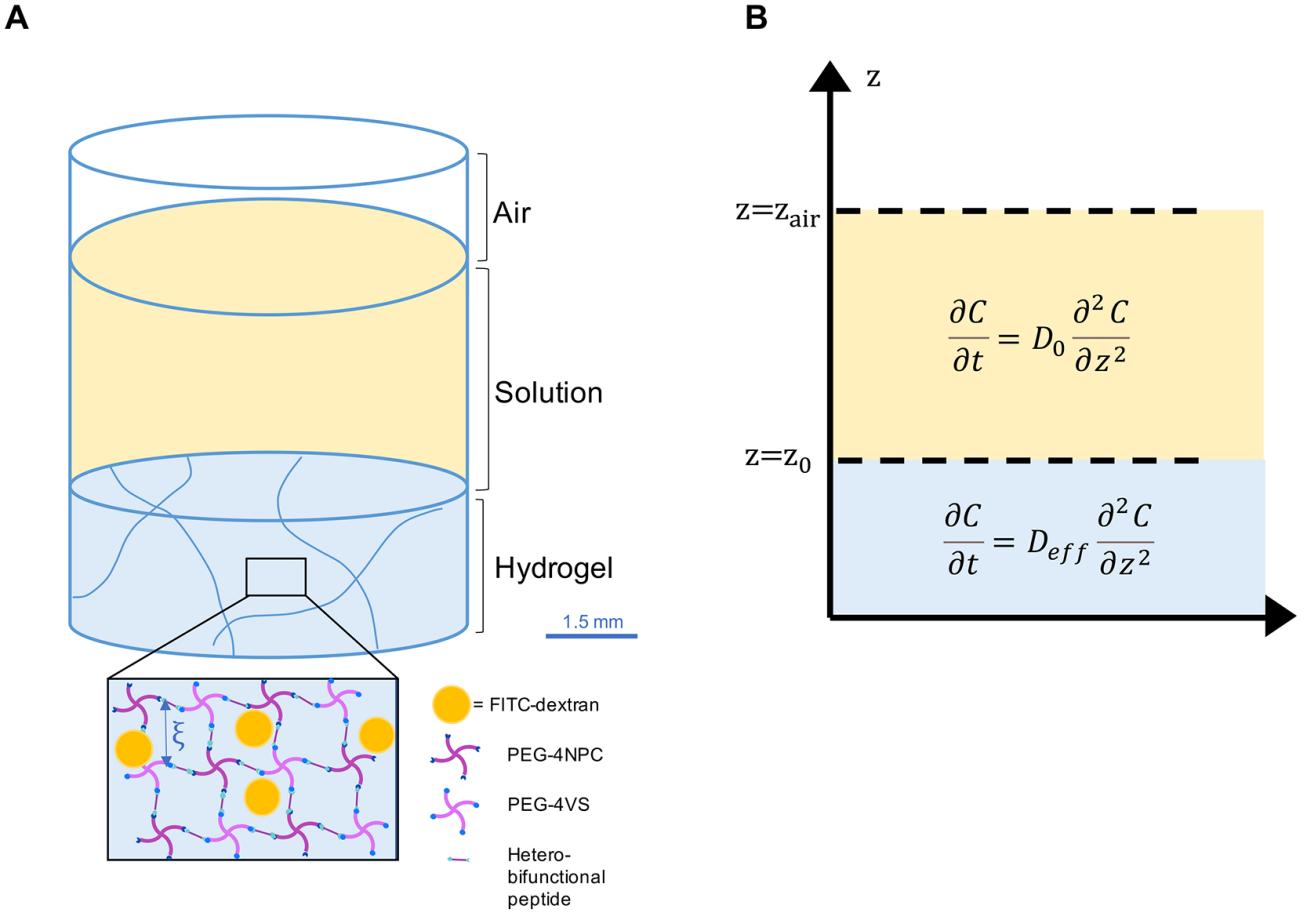
(A) Experimental setup showing the two regions through which mass transport of a solute of a given size is mathematically modeled. (B) Schematic representation of the corresponding mathematical model setup in A. Free diffusion is prescribed in the solution region with reduced diffusivity in the hydrogel modeled by altering the diffusion coefficient.

Our model assumed the hydrogel to be a homogeneous porous network of fixed mesh size ξ, using theoretical estimates calculated for A_4_+B_4_ hydrogels with polymer concentrations of 2.5%, 5%, and 10%, and using direct measurements of the mass swelling ratio. The hydrogel domain was modeled to be one-fifth of the height of the media domain above it, which represented a 60 μL hydrogel under 240 μL of solution in a well of a 96-well plate (Figure 2A). The diffusion of FITC-labeled dextran molecules of molecular weight 10, 40, and 70 kDa corresponding to hydrodynamic radii of approximately 2.3, 4.5, and 6 nm, respectively, was modeled for hydrogels created at all three polymer concentrations. The Fickian diffusion of a species *C*, representing the FITC-labeled dextran molecule, is described generally using the diffusion equation:
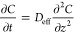
7where *C* is the species concentration in moles per cubic meter, and *D*_eff_ in square meters per second is the effective diffusion coefficient (dependent on the properties of the medium through which the species diffuses). We assumed the diffusion coefficient to be constant in space and time, assuming no spatial inhomogeneities and using parameters based on fully swollen hydrogels. We imposed free diffusion in the solution and hindered diffusion (due to the polymer network) in the hydrogel region. We imposed zero flux conditions  at the bottom of the culture well (*z* = *z*_0_) and at the media air interface (*z* = *z*_air_; Figure 2B). We prescribe continuity of the concentration and flux at the hydrogel/media interface. An initial concentration *C*_0_ was prescribed in the hydrogel region and an initial concentration of 0 in the solution phase. The diffusion coefficient in the solution is approximated as the free diffusion coefficient *D*_0_ in water of the solute and is given by the Stokes–Einstein equation:
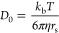
8Here, *k*_b_ is the Boltzmann constant, *T* the temperature of the solution in Kelvin, η is the dynamic viscosity of the medium in Pascal seconds, and *r*_s_ is the hydrodynamic radius of the diffusing solute in meters.^36,37^ This approach assumes diffusing molecules to be spheres moving in a continuum of solvent.^38^

The impact of the polymer phase on diffusion is modeled through an effective diffusion coefficient that captures the dependence on polymer chain radius and mesh size. The effective diffusion coefficient through the hydrogel is given by
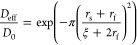
9in which *r*_f_ is the polymer chain radius and ξ the mesh size, both in meters.^35,37^ All simulations were conducted using the COMSOL Multiphysics finite element solver with the additional microfluidics module. A mesh independence study was conducted to ensure solutions’ numerical convergence as mesh element size was decreased. Elements of size 0.001 mm were used for the final simulations resulting in 50 001 elements in the mesh. The parameters in the model and their values are summarized in Table 2.

**Table 2:** Table Summarizing the Parameters Used in the Diffusion Model and Their Values

parameter	explanation	value	parameter sweep value
*k*_b_	Boltzmann constant	1.381 × 10^–23^ [m^2^ kg s^2^ K^–1^]	
*T*	absolute temperature of the solution	310.15 [K]	
η	dynamic viscosity (taken to be that of water at 37 °C)	6.913 × 10^–4^ [Pa s]	
*r*_s_	solute hydrodynamic radius	2.3 [nm] for 10 kDa FITC-dextran, 4.5 [nm] for 40 kDa FITC-dextran, 6.0 [nm] for 70 kDa FITC-dextran	2.0 [nm], 4.0 [nm], 7.0 [nm], 8.0 [nm]
*r*_f_	polymer chain radius	0.232 [nm]^33^	
ξ	mesh network size	8.41 [nm] for 2.5% hydrogels, 7.41 [nm] for 5% hydrogels, 6.43 [nm] for 10% hydrogels.	4.5 [nm], 5.0 [nm], 6.0 [nm], 7.0 [nm], 8.0 [nm], 10 [nm], 20 [nm]

### Experimental Measurements of FITC–Dextran Diffusion from Hydrogels

The 10, 40, and 70 kDa fluorescein isothiocyanate (FITC)-labeled dextran molecules (Sigma-Aldrich) were encapsulated at a concentration of 47.62 μM in 60 μL of 2.5%, 5%, and 10% hydrogels cast in flat-bottomed 96-well plates and allowed to gel for 60 min at 37 °C. Once gelled, wells were topped up with 240 μL of 30 mM HEPES buffer (pH 8). A total of 60 μL of solution was transferred to black bottom 96-well plates, and the absolute fluorescence was measured using a Promega GloMax Discover microplate reader (excitation 475 nm, emission 500–550 nm, peak emission measured). Measurements were made every hour for the first 4 h and then at regular intervals thereafter. Data were tested for significance using a one-way ANOVA with Tukey’s multiple comparison correction at 2 h and 24 h. Experimental data were then normalized to steady state fluorescence by first fitting an exponential plateau function of the form *f*(*x*) = *Y*_*m*_ × *e*^–*kx*^ where *k* represents a growth rate constant and *Y*_m_ is the maximum fluorescence value (*R*^2^ values all >0.92). Fluorescence measurements were then normalized to the end point value to enable comparisons between experimental data and modeling results. These data were then used to parametrize the model for *D*_eff_.

## Results and Discussion

### Tetra-PEG Hydrogel Physical Properties Are Dependent on Polymer Concentration

**Figure 3:**
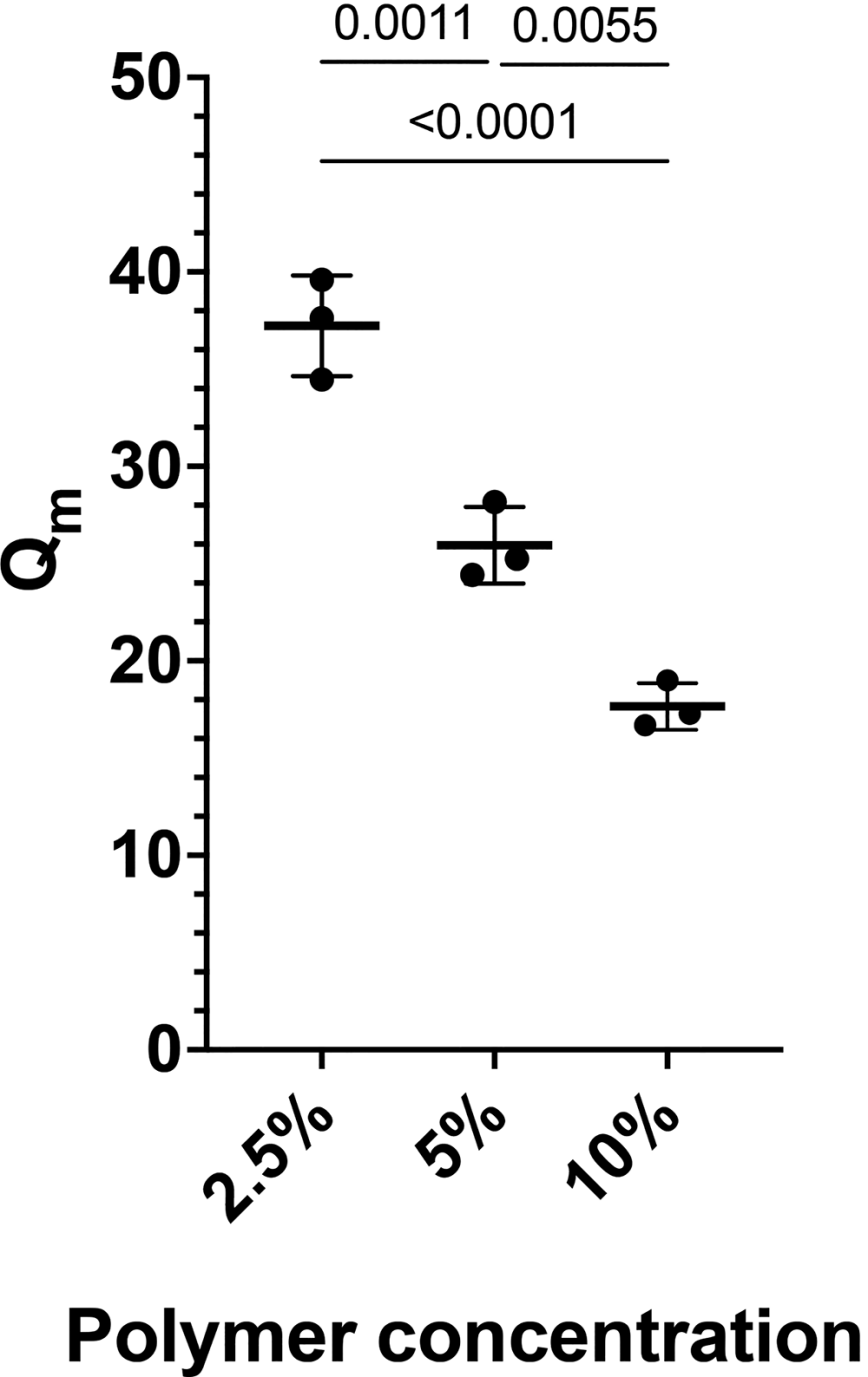
Mass swelling ratio (*Q*_m_) for 2.5%, 5%, and 10% hydrogels calculated using the hydrogels’ wet weight (at swelling equilibrium) and dry weight (*n* = 3 independent hydrogels, mean ± SD, one-way Anova with Tukey’s multiple comparison test).

To build a model of solute diffusivity, we required baseline experimental parameters for the tetra-PEG hydrogel system. Therefore, we first measured the mass swelling ratio of hydrogels with polymer concentrations of 2.5%, 5%, and 10% (Figure 3). On the basis of these values, we applied the Flory–Rehner model to calculate theoretical mesh sizes, which yielded values of 8.41, 7.41, and 6.43 nm for the 2.5, 5, and 10% hydrogels, respectively. These findings were in line with expected trends that hydrogels formed with higher polymer concentrations have smaller mesh sizes.

**Figure 4:**
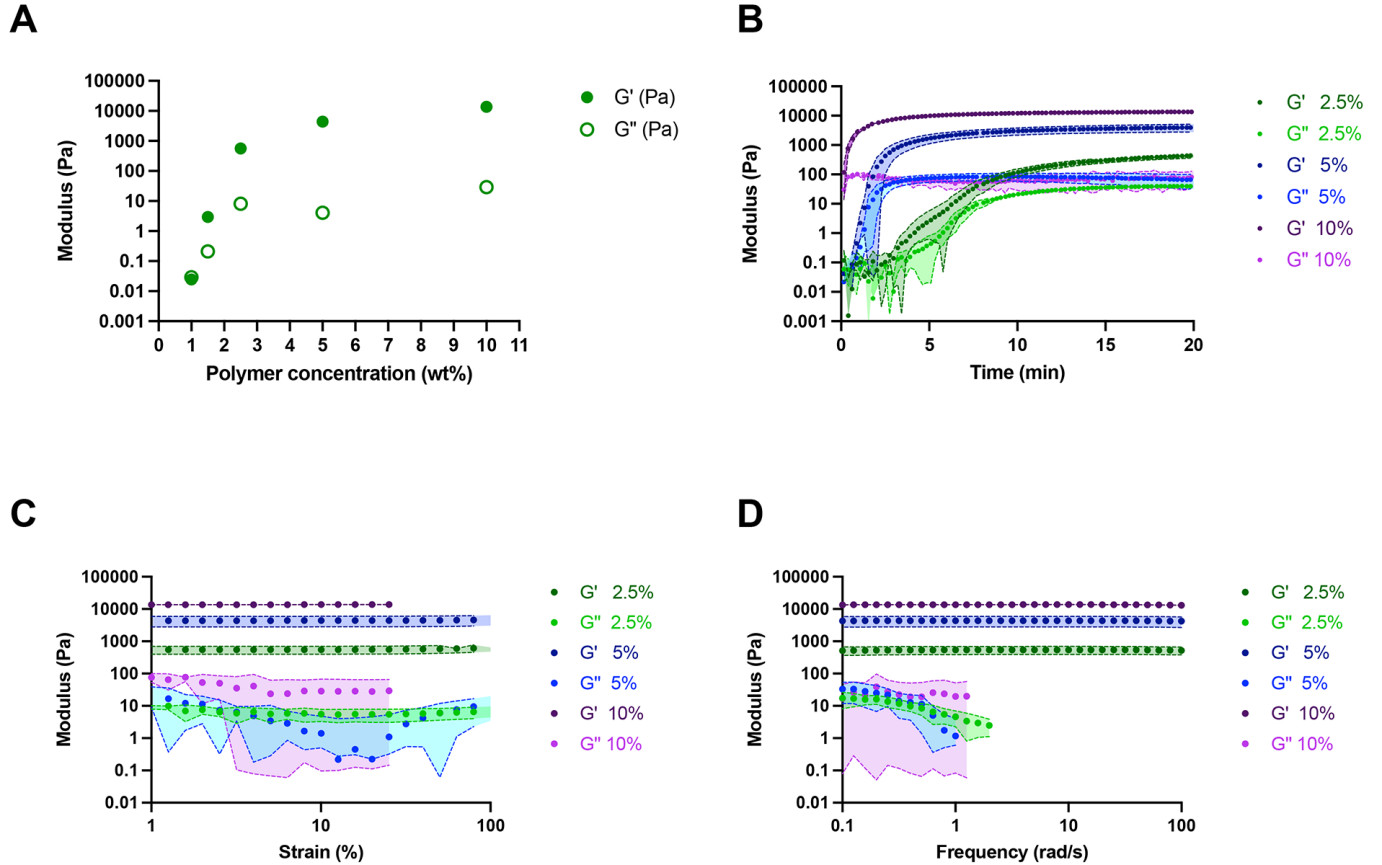
Rheological measurements performed on PEG hydrogels. (A) Mean plateau moduli of hydrogels of varying polymer concentrations. Hydrogels form (*G*′ > *G*″) at polymer concentrations ≥ 1.5%. Mean modulus was calculated from data collected for 10 min after plateau values were reached. (B) Time sweep measurements of the gelation reaction. Higher polymer concentration hydrogels have a higher plateau value and form more quickly. (C) Strain sweep measurements, *G*′ was significantly different between all three samples (*p* < 0.0001 for 2.5% vs 5%, 2.5% vs 10% and 5% vs 10%); *G*″ not significant (*p* > 0.9999 for 2.5% vs 5%, *p* = 0.2647 for 2.5% vs 10%, and *p* = 0.2659 for 5% vs 10%), both by one-way ANOVA with Tukey’s correction for multiple comparisons. (D) Frequency sweep measurements. The loss modulus could not be determined for the full range of frequencies accessed. In panels A–D, data are shown as means (dots) with the shaded area representing SD; *n* = 3 independent hydrogels for each condition. Some errors are small and not visible.

Next, we characterized the mechanical behavior of the tetra-PEG hydrogels. Mechanical studies using oscillatory rheology can provide insight into hydrogel gelation kinetics and stiffness.^39^ To determine the critical polymer concentration for hydrogel formation, we tested hydrogels formed with varying polymer concentrations and determined gelation from the point at which the storage modulus (*G*′) was greater than the loss modulus (*G*″). These data show that tetra-PEG hydrogels form at polymer concentrations of 1.5% and higher (polymer concentrations of 1% behave as viscous liquids; Figure 4A). Time sweep measurements further revealed that gelation occurs more quickly for higher polymer concentration gels. Ten-percent hydrogels formed in the short time frame between loading the sample and measuring the first data point. Alternatively, 5% hydrogels reached plateau values of *G*′ and *G*″ within 10 min, and 2.5% hydrogels reached plateau values in ∼20 min (Figure 4B). These findings are consistent with theoretical predictions that an increased concentration of reactive groups should drive faster reaction kinetics. We also found that *G*′ was significantly different for all three polymer concentrations (*p* < 0.0001 for 2.5% vs 5%, 2.5% vs 10%, 5% vs 10%). However, the loss moduli did not differ significantly from one another (*p* > 0.9999 for 2.5% vs 5%, *p* = 0.2647 for 2.5% vs 10%, and *p* = 0.2659 for 5% vs 10%). The 10% hydrogel showed strain resistance up to 25%, whereas both the 5% and 2.5% polymer concentrations showed strain resistance within the accessed range (Figure 4C). No frequency dependence in storage moduli was observed for any of the three formulations (Figure 4D). Taken together, these data show that tetra-PEG hydrogels form at polymer concentrations ≥ 1.5% and that their gelation kinetics and resulting equilibrium moduli follow expected patterns based on polymer concentrations.

### Mathematical Models Predict That Mesh Size Plays a Limited Role in Diffusivity for Small Solutes

With the hydrogels’ physical properties well characterized, we next aimed to build a diffusion model treating the polymer chains as an obstruction to diffusing molecules. The hydrodynamic radii of biologically relevant proteins are generally within the range of a few nanometers. Indeed, cytokines such as IFNγ and TNFα are reported to have hydrodynamic radii of 1.85 nm^40^ and 3 nm,^41^ respectively. Bovine serum albumin is reported to be 3.56 nm^42^ and MMP-9 4.5 nm.^43^ Some secreted proteins, however, have hydrodynamic radii that are considerably larger. For example, the ubiquitous iron-storing protein ferritin has a hydrodynamic radius of 7.17 nm,^44^ and the ECM protein fibronectin is 8.7 nm.^45^ Large proteoglycans can be as much as an order of magnitude larger (∼80 nm^46^), but these are generally accepted to not diffuse within hydrogels. Therefore, we considered solutes with hydrodynamic radii of 2.3, 4.5, and 6 nm, which correspond to the predicted values for 10 kDa, 40 kDa, or 70 kDa FITC-labeled dextran.

**Figure 5:**
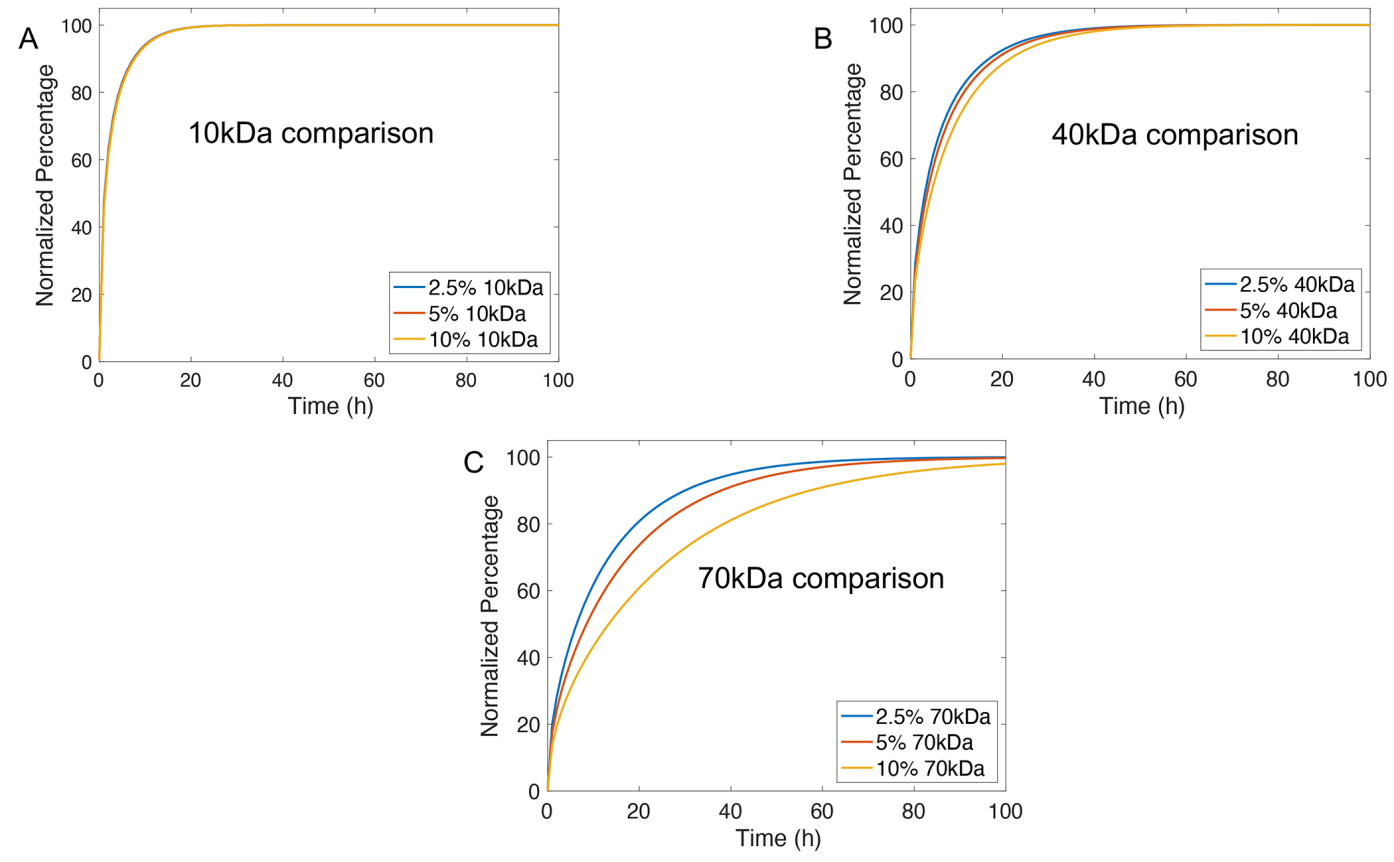
Plots generated using mathematical models showing the predicted average concentration of (A) 10 kDa, (B) 40 kDa, and (C) 70 kDa solutes in the solution above the gel normalized to the steady state concentration (to calculate a percentage of endpoint concentration) for 2.5%, 5%, and 10% hydrogels.

Using our model, we predicted the average concentration of 10 kDa, 40 kDa, or 70 kDa solutes over time in the solution above the hydrogel normalized relative to the steady state concentration prediction (Figure 5). We found that in the 10 kDa condition, diffusivity was negligibly impacted by changes in mesh size with a maximum percentage difference between the 2.5% and 10% conditions of 4.6% (Table 3). Changes in diffusivity in the 40 kDa condition were more pronounced between different mesh sizes with the largest difference of 20.8%. For the 70 kDa condition, mesh size had the greatest impact, as in 10% hydrogels we found that diffusivity changed up to 46% between profiles at each time point. In all conditions, models predicted that changing polymer concentration from 2.5% to 5% only resulted in a maximum change in diffusivity of 16%.

**Table 3:**
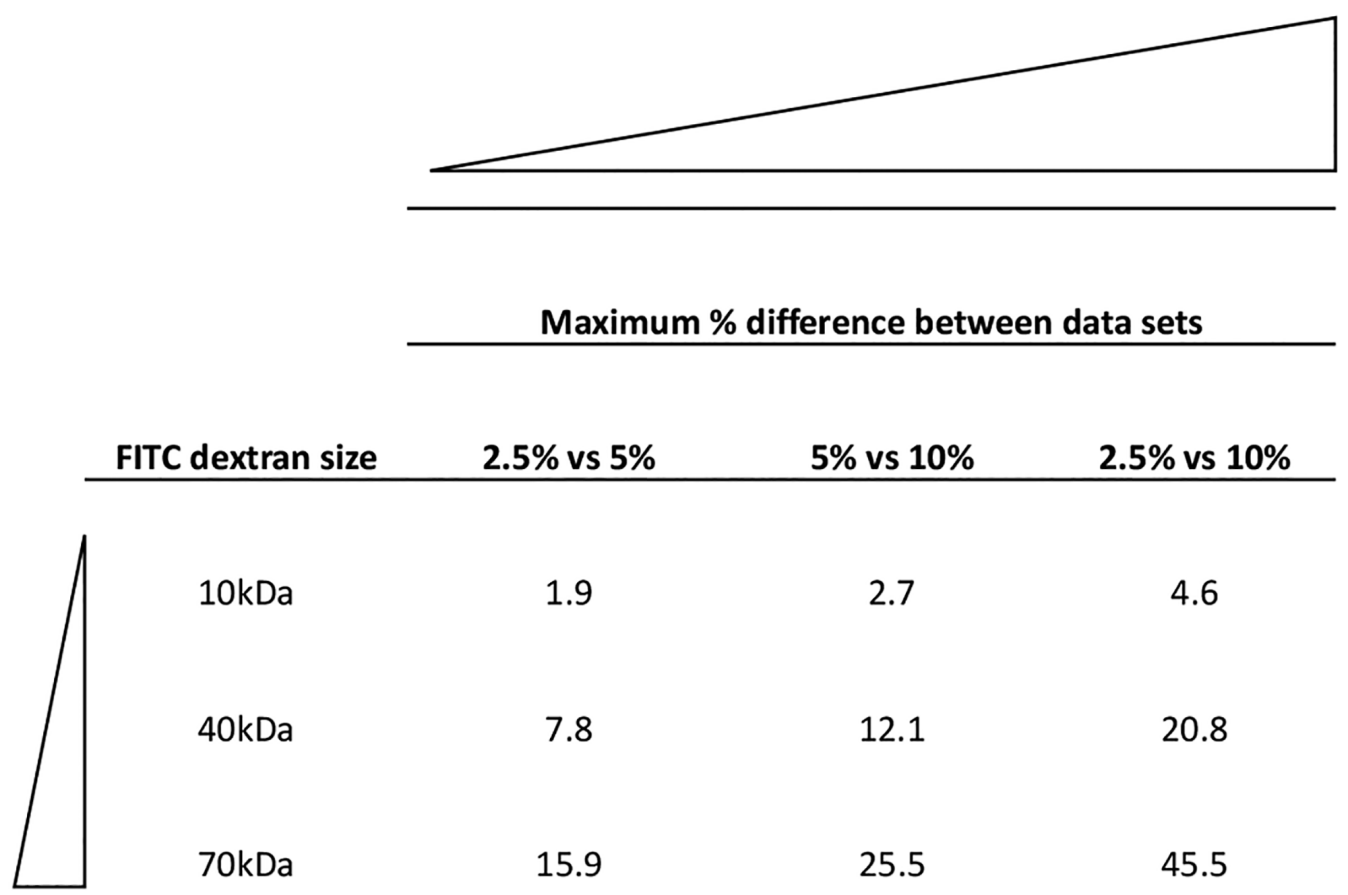
Table Summarizing the Maximum Percentage Difference between Diffusion Profiles As Predicted by Modeling Results for All Solute Sizes and Hydrogel Compositions

**Figure 6:**
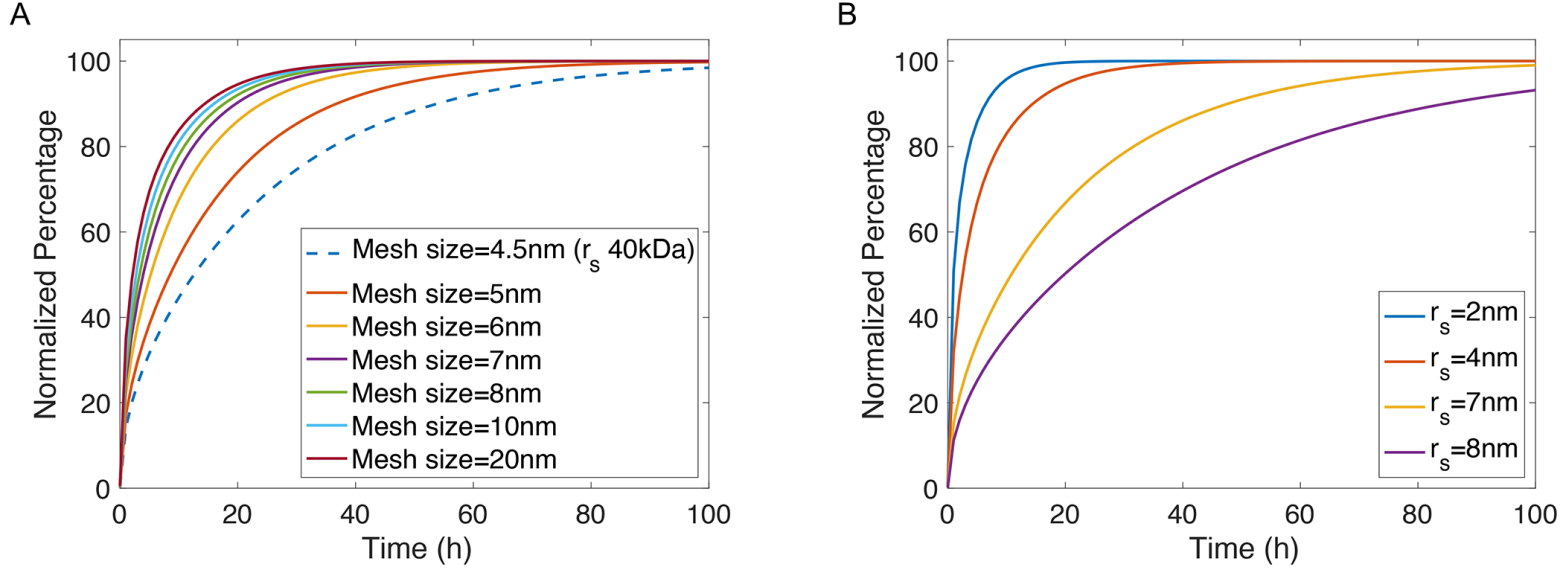
Plots generated using mathematical models showing results of parameter sweeps with (A) showing an average concentration of 40 kDa solute in the solution normalized to the steady state concentration above the hydrogel (to calculate a percentage of endpoint concentration) as the mesh network size is altered. (B) Average concentration of solute in the solution above a 2.5% hydrogel for solutes with different hydrodynamic radii (*r*_s_). As the solute size increases, the diffusivity is reduced.

The time taken to reach a steady state concentration for all hydrogel compositions for the 10 kDa condition was ∼20 h and for 40 kDa solutes was ∼50 h. However, for 70 kDa solutes, the smaller mesh size of 10% hydrogels impacted the time to a steady state, extending it beyond the ∼100 h found for the 5% and 2.5% conditions. For the 10 kDa solute, the transition to a steady state was faster compared to that of the 40 kDa solute, as half the steady state concentration was reached in <2 h. For the 40 kDa solute, this took <5 h. We then carried out parameter sweeps of solute size from 2 to 8 nm for a fixed mesh size and found that the time to achieve steady state concentration increased dramatically with increasing solute hydrodynamic radius (Figure 6). In short, our model predicts that in tetra-PEG/A_4_+B_4_ hydrogels, mesh size does not have a large influence on the diffusion of small solutes but can have a more dramatic influence on larger solutes.

### Experimental Measurements Confirm That Polymer Concentration Only Minimally Impacts Solute Diffusion

**Figure 7:**
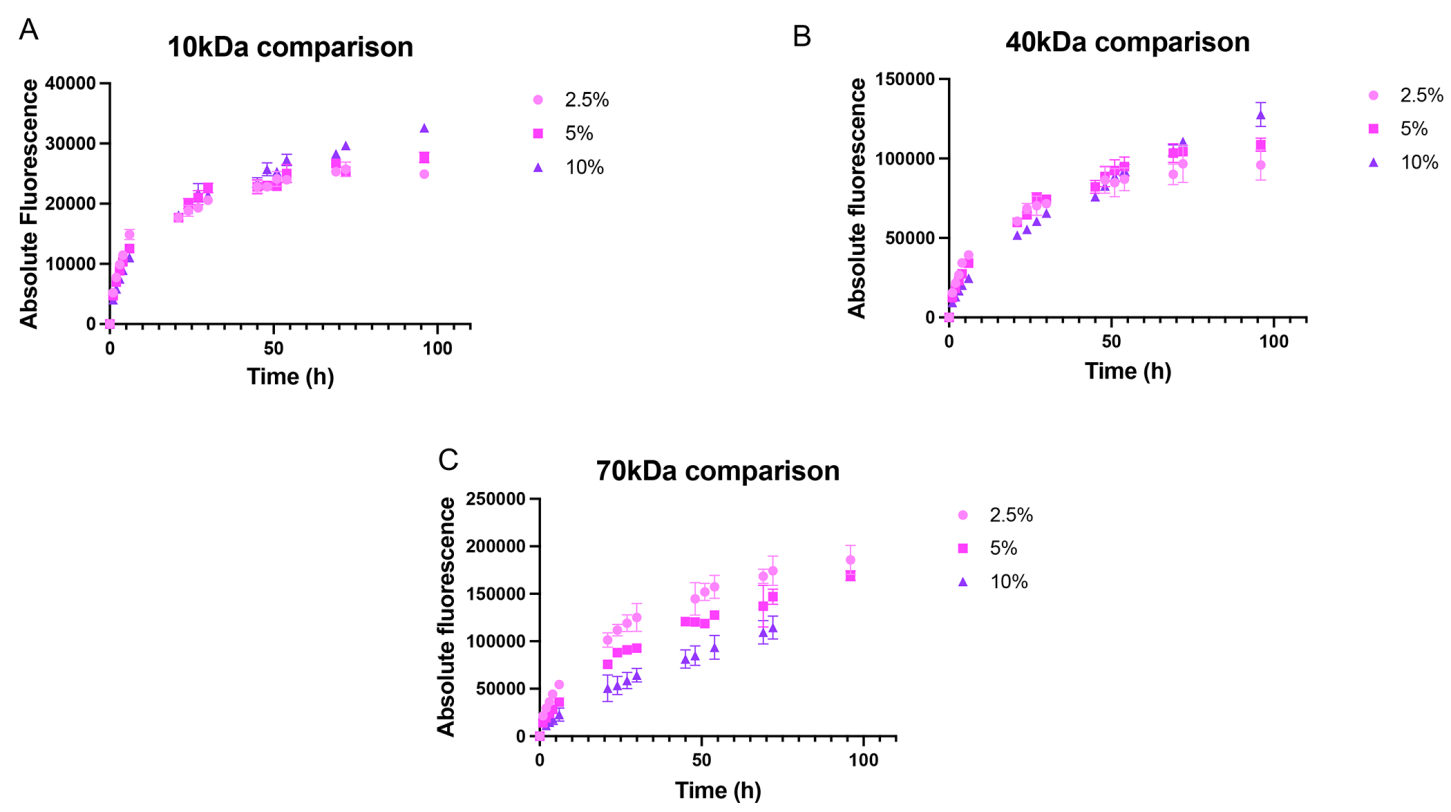
Plots showing absolute fluorescence in the media surrounding 2.5%, 5% and 10% hydrogels containing (A) 10 kDa, (B) 40 kDa, and (C) 70 kDa solutes (*n* = 3, mean ± SD). Some error bars are small and not visible.

As our model had predicted that polymer concentration only substantially impacted diffusivity in the 10% condition for the larger solute, we next aimed to measure diffusivity experimentally. We formed 2.5%, 5%, and 10% tetra-PEG hydrogels that contained 10 kDa, 40 kDa, or 70 kDa FITC-labeled dextran molecules. Broadly, we observed that smaller molecules were not differentially hindered from diffusing over 100 h, with larger differences observed between polymer concentrations for the largest molecule. In the 10 kDa condition, the diffusivity profiles for all polymer concentrations were similar, with the time taken to plateau of ∼45 h (Figure 7). Similarly, in the 40 kDa condition, 2.5% and 5% hydrogels behaved similarly, with a time taken to plateau of ∼100 h; however, the 10% profile appeared to be marginally slowed. For 70 kDa solutes, differences between polymer concentration were more apparent, again confirming that changes in mesh size have a larger effect for larger molecules (Figure 7). Statistical analyses comparing fluorescence values in the solution surrounding hydrogels after 2 h revealed significant differences between polymer concentrations for all solute sizes (10 kDa: 2.5% vs 5% *p* = 0.0035, 5% vs 10% *p* = 0.0275, 2.5% vs 10% *p* = 0.0002; 40 kDa: 2.5% vs 5% *p* = 0.0009, 5% vs 10% *p* < 0.0001, 2.5% vs 10% *p* < 0.0001; 70 kDa: 2.5% vs 5% *p* = 0.0072, 5% vs 10% *p* = 0.0360, 2.5% vs 10% *p* = 0.0005). However, by 24 h, no significant differences were detected between polymer concentration in the 10 kDa condition. In the 40 kDa condition, we detected higher levels of fluorescence in the 5% and 2.5% conditions compared to the 10% (5% vs 10% *p* = 0.0056, 2.5% vs 10% *p* = 0.0008), but the 2.5% and 5% conditions were no different. For the 70 kDa condition, we detected significant differences between polymer concentrations for all comparisons (2.5% vs 5% *p* = 0.0142, 5% vs 10% *p* = 0.0022, 2.5% vs 10% *p* = 0.0001). These observations suggest that the diffusion of larger solutes is far more impacted by changing polymer concentration than that of smaller solutes.

Overall, these data align with trends predicted by our models. They also suggest that doubling the polymer concentration from 2.5 to 5% produces hydrogels with faster gelling kinetics that are an order of magnitude stiffer without greatly affecting diffusivity in the long term. Indeed, increased polymer concentration only appears to affect the diffusion of the largest FITC-dextran molecule at stiffnesses that may not be suitable for cell encapsulation (*G*′> ∼ 10 kPa). These findings alleviate potential concerns surrounding PEG hydrogels that changes in stiffness may impact mass transport. Moreover, our hydrogel system allows us to analyze these impacts independently, thus taking advantage of the tunability of the PEG system while minimizing possible confounding effects from changes in mass transport.

Our experimental and modeling findings both identified that smaller solutes diffuse faster than larger in our tetra-PEG network. Our findings also show that the time required to achieve a steady state is non-negligible. This is of importance for 3D cell cultures in which both delivery of factors to cells from outside the hydrogel (growth factors, e.g.) and detection of biomolecules produced by cells (cytokines, e.g.) in the surrounding media depend on diffusion. In particular, the latter should only be sampled (or interpreted) at time scales that account for these effects. Furthermore, our modeling and experimental data suggest that differences between diffusion profiles tend to occur within the first hours. We suggest that differences in diffusion over this time frame are likely to have a minimal impact on experimental setups, as time scales are often longer.

**Figure 8:**
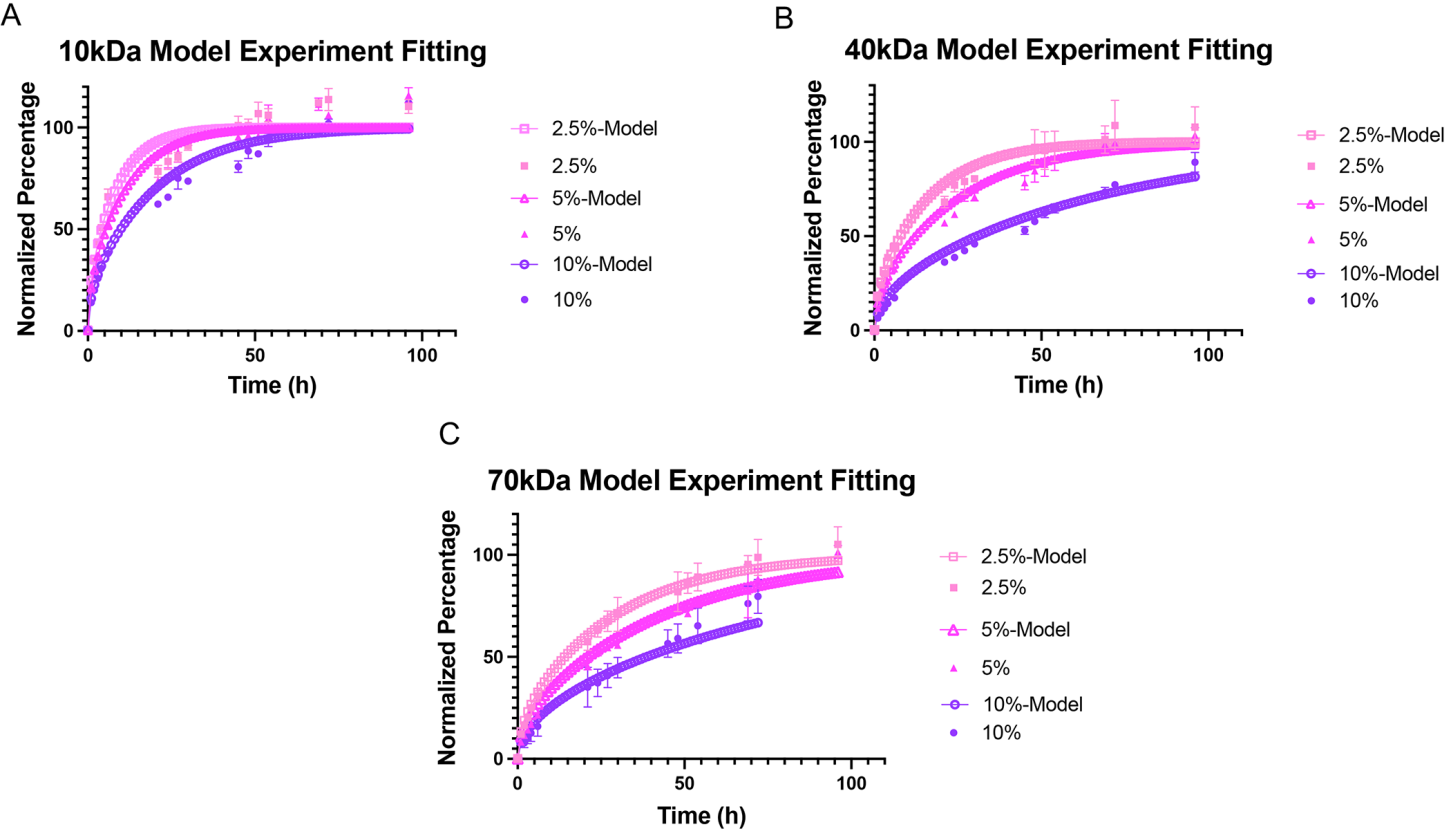
Normalized fluorescence from experimentally acquired measurements of (A) 10 kDa, (B) 40 kDa, and (C) 70 kDa FITC-labeled dextran plotted with fitted mathematical model predictions of solutes diffusing out of 2.5%, 5%, and 10% hydrogels. Experimental values are fitted to an exponential plateau function and normalized to the end point fluorescence to calculate a percentage of the total fluorescence for each time point (*n* = 3, mean ± SD). Some error bars are small and not visible.

We fitted our model to experimental results for *D*_eff_, which depends on both solute size and mesh size (*R*^2^ values all > 0.944; Figure 8). In some cases, differences between the polymer concentrations normalized versus experimental fluorescence values appeared more pronounced in models; however, this was likely attributable to our strategy of normalizing experimental data to a predicted steady state value from the data fitting. To determine which parameter plays the larger role in determining diffusivity, we analyzed the sensitivity of the model to both parameters. Our results show that network diffusivity, as initially predicted by our model, was systematically overestimated. This is in agreement with others’ findings that obstruction theory overestimates diffusivity.^37^ Such overestimations may extend from model assumptions, including that solutes are treated as hard spheres of fixed radius. In reality, molecules like dextran have more nebulous structures, and their hydrodynamic radii are unlikely to remain fixed as they diffuse through the network.^25^ Furthermore, our model predictions rely on estimates of mesh size. Mesh sizes are notoriously complex to obtain, and there remains debate concerning the accuracy of different prediction methods.^23^ A final limitation of our model is that potential interactions between the polymer and solute molecules is not accounted for; however, others have shown that this simplification is reasonable.^35,37^ Nevertheless, despite discrepancies between our predictions and experimental findings, we can have confidence in the trends predicted by our model as fitting only served to scale the value of the effective diffusivity rather than change the predicted diffusion profile.

By performing parameter sweeps changing for mesh size and solute size incrementally, we were able to observe the role of each parameter in determining overall diffusivity. Thus, our model provided additional insight into mechanisms that drive network diffusivity. Indeed, the impact of changing mesh size was negligible and only had an increasingly larger effect as mesh size approached the hydrodynamic radius of the solute. These results are in line with our experimental findings that increasing polymer concentration to 10% had a greater impact on diffusion compared to increasing from 2.5% to 5%. On the other hand, our models predicted that solute size played a far larger role in predicting diffusion, with larger solutes taking longer to diffuse than smaller. For example, our model predicted that solutes with a radius of 7 nm versus 8 nm diffusing out of a 2.5% hydrogel produced a maximum percentage difference between profiles of 36%.

Our theoretical estimates predict that 40 kDa and 70 kDa molecules should remain trapped within hydrogels, as we predicted hydrogel mesh size to be smaller than dextran molecules’ hydrodynamic diameter. However, though diffusion was increasingly hindered at higher polymer concentrations for larger molecules, they were still able to escape the hydrogels, in keeping with previous reports.^25^ These findings suggest an underestimation of mesh size or an overestimation of solute effective radius. It is also possible that encapsulating FITC-dextran within hydrogels impacts the mesh, driving the inconsistencies. In short, while theoretical predictions are useful for estimating diffusivity, these discrepancies highlight the importance of experimentally measuring diffusion. Parameters from these experiments can then be used to improve models.

## Conclusions

The diffusivity of solutes in hydrogels is important for the viability and activity of encapsulated cells and will regulate the diffusion and/or local retention of secreted factors, which play important roles in regulating cell behavior.^22,47,48^ Here, we show using theoretical estimates of hydrogel mesh size that predictions for the diffusivity of solutes with known hydrodynamic radii reasonably match experimental diffusion behaviors. We also show that altering polymer concentration in our tetra-PEG design allows us to produce hydrogels with different mechanical stiffnesses without significantly impacting diffusivity for hydrogels up to a polymer concentration of 5%. Hydrogels are increasingly used to explore hypotheses regarding the role of mechanical stiffness in regulating cell behaviors. Our findings provide confidence that such questions can be addressed in tetra-PEG hydrogels without introducing the confounding effect of differing transport rates of solutes to and from encapsulated cells. Moreover, as the A_4_+B_4_ hydrogel design allows for stiffer hydrogels to be formed at low polymer concentrations compared to A_2_+B_4_ designs, stiffer *in vitro* tissue models can be formed without compromising diffusivity. However, our findings also highlight the importance of solute size on diffusion rates, suggesting that it should be an important consideration in experimental designs that aim to deliver factors to encapsulated cells or assay secreted proteins in culture supernatants. Combined with our previous work^11^ showing that tetra-PEG designs can also allow for quick gelation at low polymer concentrations, producing hydrogels that are sufficiently soft for encapsulation of human intestinal organoids, we have gone some way to demonstrate that these hydrogels are suitable for a range of applications.

## Notes

According to UK research councils’ Common Principles on Data Policy and the Wellcome Trust’s Policy on data, software and materials management and sharing, all data supporting this study is available within the manuscript.
